# Density dependence across multiple life stages in a temperate old-growth forest of northeast China

**DOI:** 10.1007/s00442-012-2481-y

**Published:** 2012-10-02

**Authors:** Tiefeng Piao, Liza S. Comita, Guangze Jin, Ji Hong Kim

**Affiliations:** 1 Center for Ecological Research, Northeast Forestry University, Harbin, 150040 China; 2 College of Forest and Environmental Sciences, Kangwon National University, Chuncheon, 200-701 Korea; 3 Department of Evolution, Ecology and Organismal Biology, The Ohio State University, Columbus, OH 43210 USA; 4 Smithsonian Tropical Research Institute, Box 0843-03092, Balboa, Ancón, Republic of Panamá; 5 Present Address: College of Forest and Environmental Sciences, Kangwon National University, Chuncheon, 200-701 Korea

**Keywords:** Species coexistence, Janzen–Connell hypothesis, Liangshui FDP, *Pinus koraiensis*, Habitat heterogeneity

## Abstract

Recent studies on species coexistence suggest that density dependence is an important mechanism regulating plant populations. However, there have been few studies of density dependence conducted for more than one life-history stage or that control for habitat heterogeneity, which may influence spatial patterns of survival and mask density dependence. We explored the prevalence of density dependence across multiple life stages, and the effects of controlling for habitat heterogeneity, in a temperate forest in northeast China. We used generalized linear mixed-effects models to test for density-dependent mortality of seedlings and spatial point pattern analysis to detect density dependence for sapling-to-juvenile transitions. Conspecific neighbors had a negative effect on survival of plants in both life stages. At the seedling stage, we found a negative effect of conspecific seedling neighbors on survival when analyzing all species combined. However, in species-level analyses, only 2 of 11 focal species were negatively impacted by conspecific neighbors, indicating wide variation among species in the strength of density dependence. Controlling for habitat heterogeneity did not alter our findings of density dependence at the seedling stage. For the sapling-to-juvenile transition stage, 11 of 15 focal species showed patterns of local scale (<10 m) conspecific thinning, consistent with negative density dependence. The results varied depending on whether we controlled for habitat heterogeneity, indicating that a failure to account for habitat heterogeneity can obscure patterns of density dependence. We conclude that density dependence may promote tree species coexistence by acting across multiple life-history stages in this temperate forest.

**Electronic supplementary material:**

The online version of this article (doi:10.1007/s00442-012-2481-y) contains supplementary material, which is available to authorized users.

## Introduction

Understanding the mechanisms of population size regulation is of vital importance in the study of species coexistence and biodiversity maintenance. Recent studies have provided strong evidence that density-dependent processes play a role in shaping plant communities (Wills and Condit 1999; Harms et al. 2000; Hille Ris Lambers et al. 2002; Comita et al. 2010). Density-dependent mortality and growth can be generated by intraspecific competition for resources (Wright 2002). In addition, since Janzen (1970) and Connell (1971) reported that host-specific natural enemies reduce survival when a species occurs at high local densities, specialized herbivore and pathogen-induced negative density dependence has also been considered a potentially important mechanism regulating population dynamics and facilitating species coexistence in diverse tree communities (Wright 2002).

Numerous studies have examined the importance of density dependence in forests. For example, Harms et al. (2000) found widespread negative density dependence over the seed-to-seedling transition for 53 species on Barro Colorado Island (BCI), Panama. Metz et al. (2010) found a strong negative impact of conspecific seedling densities and adult abundance on first-year seedling survival in a study of 163 species in a lowland Amazonian rain forest. Yamazaki et al. (2009) in their study of eight tree species of a temperate forest found that six of them showed distance- and/or density-dependent seedling mortality caused by diseases and rodents. Comita and Hubbell (2009) tracked established seedlings of 235 species in the BCI 50-ha forest dynamics plot (FDP) over 3 years and also found negative effects of conspecific neighbors on survival. Together, these and additional studies (e.g. Webb and Peart 1999; Hille Ris Lambers et al. 2002; Queenborough et al. 2007; Pigot and Leather 2008) provide evidence for an important role of negative density dependence at early life stages.

Negative density dependence has also been detected at later life stages in tree communities. For example, Stoll and Newbery (2005) studied the growth of medium-sized (10 to <100 cm diameter at breast height, dbh) trees of ten abundant overstory dipterocarp species and found strong negative effects of neighbors on their growth in a lowland forest in Borneo. Similarly, Zhang et al. (2009) found tree survival was negatively correlated with conspecific basal area for 8 of 13 focal species with dbh ≥ 1 cm in the temperate forest of Changbaishan Mountain, China. Thus, previous studies indicate that negative density dependence exists at both early and later life-history stages of trees. Therefore, to test the prevalence of density dependence in a community, we must consider multiple size classes. Mortality patterns in seedlings can usually be analyzed by direct observation, due to their high mortality rates caused by susceptibility to natural enemies and environmental stressors. For larger trees that have lower mortality rates and can have a lifespan of several hundreds of years, however, several years of observation is likely too short to detect effects of density dependence (Ratikainen et al. 2008). However, if we can assume that populations of larger trees are in an equilibrium stage, spatial point pattern analysis can be an effective approach for the detection of lagged effects of density dependence, by looking at changes in aggregation of each species from early to later life-history stages. This is possible because pollen and seed dispersal limitation, which are quite common in plant communities (e.g., Hubbell et al. 1999, Cázares-Martínez et al. 2010), may cause spatial aggregation in recruitment (Wright 2002). If there is strong negative density dependence, the degree of conspecific aggregation will decline with increasing size class, due to lower survival of individuals growing in high density patches of conspecifics (Sterner et al. 1986; Barot et al. 1999; Condit et al. 2000).

One confounding factor in the analysis of density dependence is habitat heterogeneity, since a species will tend to perform better when growing in its preferred habitat (Getzin et al. 2008; Murrell 2009). If a species has high local density in its preferred habitat and low local density in marginal habitats, and host-specific natural enemies or intraspecific competition do not offset the habitat advantages, a positive relationship between conspecific density and performance would be found, despite underlying negative density-dependent effects. Therefore, tests for density dependence must account for habitat heterogeneity. However, this is made difficult by the fact that numerous environmental covariates are difficult to quantify (He and Duncan 2000; Wiegand et al. 2007). Getzin et al. (2008) developed a simple method to solve this problem. By using adult trees as “controls”, they factored out habitat heterogeneity and were able to detect conspecific density-dependent thinning in western hemlock populations.

In this study, we explore the prevalence of density dependence across multiple life stages in a temperate forest of northeast China. We use data on 5,762 seedlings of 34 woody species to examine the effects of conspecific density on the survival of established seedlings (early life-history stage). In addition, we use data on 15 abundant woody species with dbh ≥ 1 cm (later life-history stages) to examine conspecific density-dependent thinning from sapling to juvenile stages using spatial point pattern analysis. For all life-history stages studied, we accounted for habitat heterogeneity that may mask density-dependent effects.

We test the hypothesis that density dependence is prevalent both in early and later life-history stages of trees. Specifically, for seedlings, we test the hypothesis that survival in the seedling bank declines with increasing local conspecific neighbor density and the effect of conspecific neighbors differs from that of heterospecifics. For larger trees, we test the hypothesis that the extent of aggregation declines from sapling to juvenile stages, indicating the existence of negative density dependence at later life-history stages.

## Materials and methods

### Study site and data collection

Our study site, termed Liangshui forest dynamics plot (FDP) is located in Liangshui national reserve (47°10′50″N, 128°53′20″E) of northeastern China (Online Resource 1). The reserve is characterized by rolling mountainous terrain with elevation ranging from 280 to 707 m. a.s.l. Mean annual temperature is −0.3 °C with mean daily maximum temperature of 7.5 °C and minimum temperature of −6.6 °C. Mean annual surface soil temperature is 1.2 °C with 100–120 frost-free days. Mean annual precipitation is 676 mm with 78 % relative humidity and an evaporation rate of 805 mm (Jin et al. 2006).

The 9-ha (300 × 300 m) FDP was established in 2005 in a typical mixed broadleaved-Korean pine (*Pinus koraiensis*) forest. All woody stems ≥2 cm dbh in the plot were mapped, measured, identified to species, and tagged in 2005. In 2010, we recensused the plot and additionally included woody stems 1–2 cm dbh. In the 2010 census, we documented 21,775 free-standing live individuals ≥1 cm dbh belonging to 18 families, 32 genera and 46 species (species identifications based on Chou et al. 1985). In this paper, we used data on live trees ≥1 cm dbh from the 2010 census. These trees were grouped by maximum attainable height into five growth forms: shrubs (S; ≤5 m), small understory tree species (US; 5 to ≤10 m), large understory tree species (UL; 10 to ≤20 m), small canopy tree species (CS; 20 to ≤30 m) and large canopy tree species (CL; >30 m). In turn, each growth form was divided into three dbh size classes to define life-history stages: sapling, juvenile, and adult stages (Table 1). For analyses with trees ≥1 cm dbh, 15 species that had ≥40 individuals at each of these life stages were selected as focal species (Online Resource 2). These species comprised 89 % of total stems ≥1 cm dbh in the plot.

**Table 1:** Life stage classifications based on dbh (cm) for trees of different growth forms: shrubs (*S*), small understory tree species (*US*), large understory tree species (*UL*), small canopy tree species (*CS*), and large canopy tree species (*CL*), respectively

Life stage	S	US	UL	CS	CL
Sapling	1.0−1.5	1.0−2.0	1.0−2.5	1.0−5.0	1.0−8.0
Juvenile	1.6−2.0	2.1−4.0	2.6−6.0	5.1−10.0	8.1−15.0
Adult	>2.0	>4.0	>6.0	>10.0	>15.0

Dbh cut-offs were selected to ensure adequate sample sizes for the spatial point pattern analysis

In 2005, we established a permanently marked 4-m^2^ seedling plot in the northwest corner of each 10 × 10 m subplot of the 9-ha FDP. All free-standing, woody seedlings and small saplings ≥30 cm tall and <1 cm dbh (hereafter referred to as seedlings) were tagged, measured, and identified to species within each seedling plot. We recensused the 900 seedling plots in 2007, 2008, and 2010.

To estimate light conditions above the seedling plots, hemispherical photographs were taken using a fisheye lens (Nikon FC-E8) mounted on a Nikon camera (Coolpix 4500) at a height of 1.3 m over the center of each seedling plot during August 2005. We analyzed the hemispherical photos using Hemiview canopy analysis software v.2.1 (Delta-T De-vices, UK, 1999) to calculate the percent canopy openness above each seedling plot. To investigate effects of topography, the entire study plot was first divided into 3,600 contiguous 5 × 5 m quadrats, and then the topographic position (ridge, upper slope, lower slope, and valley) for each quadrat containing a seedling plot was assessed visually by comparing the topography to surrounding quadrats.

### Data analysis

We tested for conspecific negative density dependence at two life stages: the established seedling stage (early-stage) and the sapling-to-juvenile transition (later-stage). However, because species habitat preferences may obscure underlying density-dependent processes, we first examined evidence for effects of habitat heterogeneity.

### Test of habitat heterogeneity

Spatial patterns of mature trees can be used as an indicator of strong environmental habitat preferences, under the assumption that mature trees have undergone thinning over time due to environmental filtering (Getzin et al. 2008). Although dispersal limitation can also leave a signature on the spatial pattern of trees, mature individuals likely represent those who lived in sites most favorable for the species (Condit et al. 2000) and thus their spatial patterns can be used to detect underlying habitat heterogeneity. To test for habitat preferences at our study site, we analyzed the spatial pattern of adult trees for the 15 focal species, using the cumulative *L*-function (the transformed *K*-function, $L(r) = \sqrt {\frac{K(r)}{\pi }} - r$, where *r* is the variable radius sampled around each tree of each focal species; further methodological details are explained in “Later-stage conspecific density dependence”, below) (Ripley 1976; Stoyan and Stoyan 1994; Illian et al. 2008) with the homogeneous Poisson process as a null model. Stoyan and Penttinen (2000) suggested that, in mature boreal forests, tree–tree interactions are independent at scales >10 m, and, beyond this scale, spatial patterns of trees are influenced by environmental factors. In this study, we also assumed that tree–tree interactions can be neglected beyond the scale of 10 m, and consider aggregated patterns of adult trees at scales >10 m as a sign of habitat heterogeneity.

### Early-stage density dependence

To test for density dependence at the seedling stage, we examined the effect of neighbor density on seedling survival using generalized linear mixed-effects models (GLMMs) with binomial errors. We modeled the probability of an individual seedling surviving across the 2005–2010 census intervals as a function of the density and identity of seedling and tree (≥1 cm dbh) neighbors. Local seedling densities were obtained by counting the number of conspecific (*S*_CON_) and heterospecific (*S*_HET_) seedlings in the same 4-m^2^ quadrat as the focal seedling in 2005, and local densities of trees were calculated by summing up the basal area (*B*) of all conspecific (*B*_CON_) and heterospecific (*B*_HET_) trees ≥1 cm dbh in the 2010 census within a radius of 10 m. A radius of 10 m was selected since it yielded the lowest AIC value compared with 5-, 15-, and 20-m radii in preliminary analyses using the full model (model 9 in Table 2). In addition to conspecific and heterospecific neighbor densities, initial seedling height (*H*) was included as a fixed effect, since seedling size is usually significantly and positively correlated with survival.

**Table 2:** Nine models compared to determine effects of conspecific and heterospecific neighbor densities on established seedling survival in the Liangshui FDP

Model class	Model	Model structure
Density independent	1	*a* + *b* × *H*
Effect of conspecific density = effect of heterospecific density	2	*a* + *b* × *H* + *c* × *S*_TOTAL_
	3	*a* + *b* × *H* + *c* × *S*_TOTAL_ + *d* × *B*_TOTAL_
	4	*a* + *b* × *H* + *d* × *B*_TOTAL_
Effect of conspecific density ≠ effect of heterospecific density	5	*a* + *b* × *H* + *c*_1_ × *S*_CON_ + *c*_2_ × *S*_HET_
	6	*a* + *b* × *H* + *c*_1_ × *S*_CON_ + *c*_2_ × *S*_HET_ + *d* × *B*_TOTAL_
	7	*a* + *b* × *H* + *d*_1_ × *B*_CON_ + *d*_2_ × *B*_HET_
	8	*a* + *b* × *H* + *c* × *S*_TOTAL_ + *d*_1_ × *B*_CON_ + *d*_2_ × *B*_HET_
	9	*a* + *b* × *H* + *c*_1_ × *S*_CON_ + *c*_2_ × *S*_HET_ + *d*_1_ × *B*_CON_ + *d*_2_ × *B*_HET_

*H* seedling height; *S*_*CON*_, *S*_*HET*_ and *S*_*TOTAL*_ number of conspecific, heterospecific and overall seedling neighbors of the focal seedling in the 4-m^2^ seedling plot; *B*_*CON*_, *B*_*HET*_ and *B*_*TOTAL*_ basal area of conspecific, heterospecific and overall tree neighbors (≥ 1 cm dbh) within a radius of 10 m; *a*, *b*, *c*, *c*_1_, *c*_2_, *d*, *d*_1_ and *d*_2_ model coefficients

We included seedling plot as a random effect in the model to account for spatial autocorrelation in survival. Including a plot term should be sufficient, since all seedling plots are spaced 10 m apart and spatial autocorrelation of model residuals was found to be negligible beyond 10 m (Online Resource 3). We also included species as a random effect in the community-level models, in order to allow for differences among species in their baseline survival rates (i.e. the model intercept term). We excluded *Sorbaria sorbifolia* and *Spiraea salicifolia* from all analyses, since they did not have stems of dbh ≥ 1 cm in the study plot.

To assess the role of conspecific and heterospecific neighbor densities on seedling survival, nine models were constructed according to Comita and Hubbell (2009) (Table 2). These nine models fall into three classes: (1) a density-independent model, (2) models in which there is an effect of overall seedling or tree neighbor densities, with no differentiation between conspecifics and heterospecifics, and (3) models in which the effect of conspecifics differs from heterospecifics for seedling or tree neighbors. Models were compared using Akaike’s information criterion (AIC; Burnham and Anderson 2003).

We examined the effect of neighbors on seedling survival at three levels. First, we examined seedling survival on a species-by-species basis for 11 abundant species (*n* > 99 seedlings). Second, we examined seedling survival for all species combined in the whole dataset. Third, we excluded those species that showed density-dependent mortality in the first level analysis and examined patterns of seedling survival for the remaining species combined, in order to determine whether community-wide results were being driven by a few species.

To test whether species’ habitat preferences affected our ability to detect density dependence, we repeated the above analyses with the abiotic variables of canopy openness and topography position as covariates to control for habitat heterogeneity in each of the nine models. In the species-level analyses, we included canopy openness and topography position as fixed effects. In the community-wide analyses, because we expected species to vary in their responses to canopy openness and topography position, we included the two variables as random effects that varied among species. We also ran the models using altitude (as a continuous variable) instead of the topographic categories, but the results were qualitatively similar, so we only present the results using topographic categories, since they better capture the microhabitat variation in the plot.

GLMMs were fitted by the *lmer*() function of the ‘lme4’ package in R 2.13.0 (R Development Core Team 2011) with the recommended Laplace method (Bates et al. 2008; Bolker et al. 2009).

### Later-stage conspecific density dependence

**Fig. 1:**
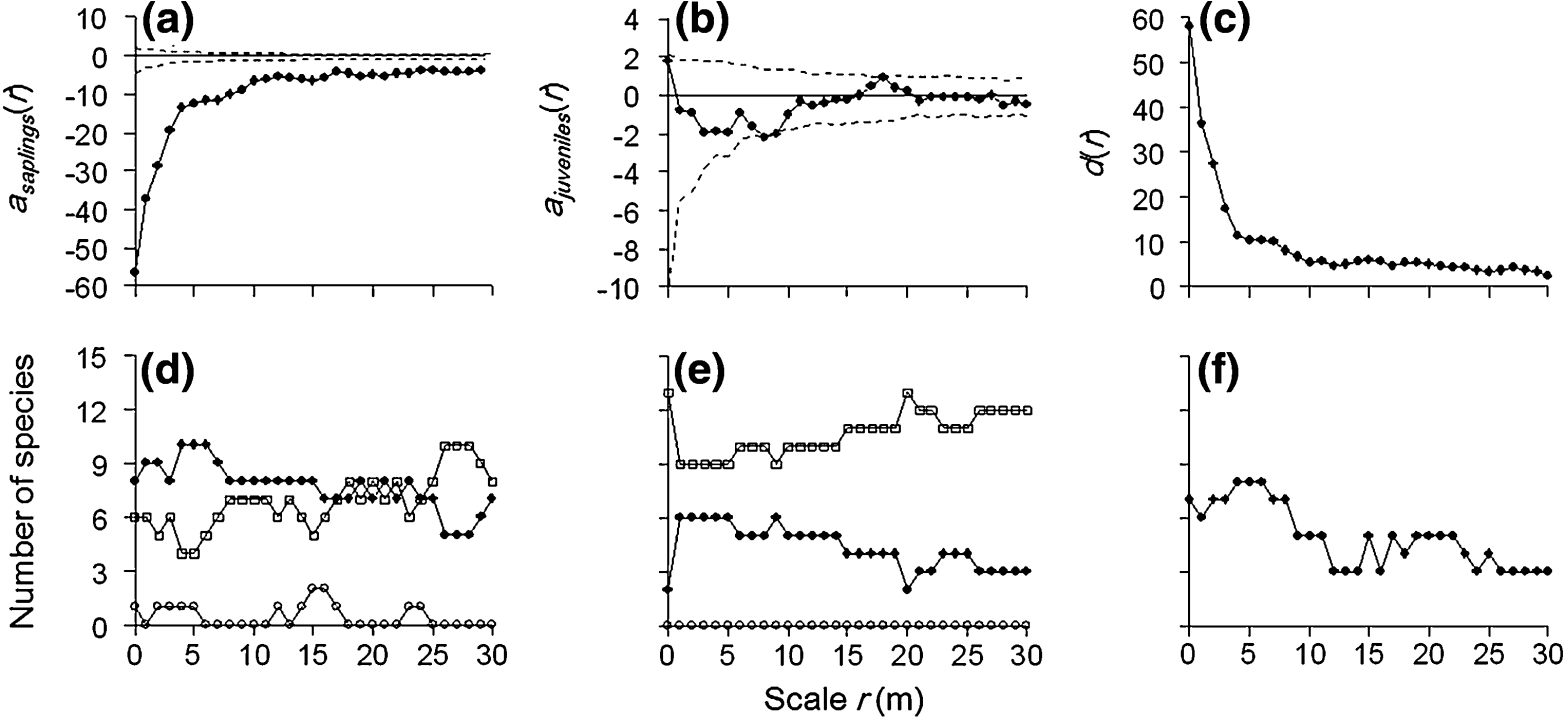
Examples of conspecific density-dependent analysis using *Pinus koraiensis* **a** saplings, **b** juveniles as cases and **c** decline of additional aggregation from the sapling to juvenile stage. The maximum strength of conspecific thinning (*d*_max_) took place at the scale of 0 m. Results for the analysis of all 15 focal species: **d** saplings, **e** juveniles as cases and **f** number of focal species showing density dependence at each scale. In (**d**) and (**e**), *solid circles* represent the number of species with the test statistic *g*_ij_(*r*) − *g*_ii_(*r*) < 0 (i.e. cases show additional aggregation relative to adults), *open circles* represent the number of species with *g*_ij_(*r*) − *g*_ii_(*r*) > 0 (i.e. cases are less aggregated than adults), and *open squares* represent the number of species with *g*_ij_(*r*) − *g*_ii_(*r*) = 0 (i.e. patterns for cases and adults are created by the same stochastic process)

For each of the 15 focal species selected for later-stage density dependence analysis, we applied the method of random-labeling null model within a case–control design (Getzin et al. 2008) to estimate conspecific density-dependent thinning from the sapling to juvenile stage, utilizing the bivariate pair correlation *g*-function (Stoyan and Stoyan 1994; Illian et al. 2008). We used saplings and juveniles as cases (pattern *i*) and adults as controls (pattern *j*) to account for habitat heterogeneity. The *g*-functions are invariant under random thinning of trees, hence we would expect $g_{ij} (r) = g_{ji} (r) = g_{ii} (r) = g_{jj} (r)$, where *r* denotes distance scale. We used $a_{i} (r) = g_{ij} (r) - g_{ii} (r)$ as a test statistic to test whether cases *i* show an additional pattern that is independent from the controls *j.* If *a*_*i*_(*r*) < 0, cases can be said to exhibit additional aggregated patterns relative to adults, irrespective of whether habitat heterogeneity is present or not (Getzin et al. 2006; Watson et al. 2007). The change in additional aggregation from sapling to juvenile stages can be expressed by the formula: $d(r) = a_{\text{juveniles}} (r) - a_{\text{saplings}} (r)$ (Zhu et al. 2010), where *a*_juveniles_(*r*) is the additional aggregation of juveniles relative to adults over the scales *r*, and *a*_saplings_(*r*) is the additional aggregation of saplings. For a particular species, if *a*_saplings_(*r*) < 0 and *d*(*r*) > 0, we would infer that conspecific density-dependent thinning takes place from cohorts of saplings to cohorts of juveniles for that species. We focused on the scale of 0–10 m for the analysis above, because we assume the effects from tree–tree interactions could be efficiently indicated by this scale, and changes in spatial pattern beyond a scale of 10 m could be caused by other environmental factors (i.e. large-scale habitat heterogeneity; Stoyan and Penttinen 2000). *d*_max_ was the maximum strength of conspecific thinning, when *d*(*r*) takes the maximal value at the scale of 0–30 m. We provide an example to illustrate this part of the analysis using *Pinus koraiensis* (Fig. 1a–c).

To assess the importance of controlling for habitat preference in the analysis of density dependence, we also additionally randomized the locations of adult trees for each species and used them as pattern *j* in place of the actual adult pattern which controls habitat preference and repeated the analysis described above.

All spatial point pattern analysis was done in the grid-based software Programita (Wiegand and Moloney 2004), using resolutions of a grid size of 1 m^2^ and a ring width of 3 m for analysis of tree–tree interactions and habitat heterogeneity at scales of 0–30 m. The resolutions were selected based on the size of our 300 × 300 m plot and the measurement uncertainty of point coordinates, and they should be sufficient to capture detailed variation in the pair-correlation function over the range of scales where we expected significant effects (effects from tree–tree interactions and habitat heterogeneity) up to 30 m (Wiegand and Moloney 2004; Zhu et al. 2010).

For all spatial point pattern analysis, we performed 999 Monte Carlo simulations of the null model and used the fifth-lowest and fifth-highest values (i.e., extreme 0.5 % simulated cases at either end) as simulation envelopes. However, because the simulation tests are performed at different scales concurrently, this simulation inference yields an underestimated Type I error rate (Loosmore and Ford 2006). We therefore combined this simulation envelope method with a goodness-of-fit test (GOF) (Diggle 2003). Further analysis were only performed for those data sets where the observed GOF’s *P* < 0.005 (Loosmore and Ford 2006; Wiegand et al. 2007).

## Results

### Test of habitat heterogeneity

Except for *Ulmus laciniata*, all focal species showed significant aggregation up to 30 m (i.e. *P* < 0.005 for GOF tests). Adults of 12 of the 15 focal species showed increasing aggregation at scales *r* > 10 m (Online Resource 4). This indicated that most of the focal species exhibited habitat preference caused by large-scale habitat heterogeneity, suggesting that we should account for habitat heterogeneity in our analysis of density dependence.

### Early-stage density dependence

For a total of 5,762 seedlings, mortality was 29.8 % from 2005–2010, thus averaging ~6 % per year. Among the 11 abundant focal species, percent seedling mortality between 2005 and 2010 ranged from 11.0 to 55.4 % (mean = 38.8 %).

Before controlling for habitat preference, for 5 of the 11 focal species, the best-fit model was the density-independent model, indicating that neither seedling nor tree neighbors influenced seedling survival. For 4 species, the best-fit model included overall seedling density or basal area of trees, with no difference between the effects of conspecifics and heterospecifics. For the remaining 2 species, the best-fit model included separate terms for conspecific and heterospecific neighbors. For *Philadelphus schrenkii*, the best-fit model (model 5) included conspecific and heterospecific seedling densities, but not tree basal area, and the effect of conspecific seedling density was significantly negative. For *Deutzia glabrata*, the best-fit model was the full model (model 9), which included separate terms for conspecific and heterospecific seedling and tree neighbors. The effect of conspecific neighbors was significantly negative for both seedling density and basal area of trees ≥1 cm dbh. In contrast, the effect of heterospecific basal area of trees ≥1 cm dbh was significantly positive (Online Resource 5). Thus, of the 11 focal species, only 2 showed patterns of seedling survival consistent with conspecific negative density dependence.

Nonetheless, in the community-wide analysis, we detected significant conspecific negative density dependence. With all species combined, the probability of seedling survival was best described by model 6, which included separate conspecific and heterospecific seedling terms and overall basal area of trees. The effect of conspecific seedling density was significantly negative. The effect of overall basal area of trees ≥1 cm dbh was significantly positive (Table 3).

**Table 3:** Effects of local-scale seedling and adult neighbor densities on survival of established seedlings with and without controlling for habitat preference in the 9-ha plot (see “Materials and methods”)

Dataset	Whole dataset (BF = 6, *n* = 5,762, SP = 34)		Subset (BF = 4, *n* = 3,987, SP = 32)	
Control for habitat preference	No	Yes	No	Yes
Parameter values (standard error)				
*H*	**0.283 (0.033)**	**0.282 (0.033)**	**0.255 (0.041)**	**0.257 (0.041)**
*S*_CON_	**−0.085 (0.038)**	**−0.098 (0.038)**	–	–
*S*_HET_	−0.014 (0.045)	−0.012 (0.045)	–	–
*S*_TOTAL_	–	–	–	–
*B*_CON_	–	–	–	–
*B*_HET_	–	–	–	–
*B*_TOTAL_	**0.106 (0.041)**	**0.098 (0.042)**	**0.090 (0.046)**	0.084 (0.046)
AIC				
Model 1	6,779.2	6,790.7	4,718.7	4,732.4
Model 2	6,780.9	6,792.3	4,720.6	4,734.4
Model 3	6,775.9	6,787.5	4,718.5	4,732.9
Model 4	6,775.1	6,787.5	4,716.9	4,731.3
Model 5	6,778.7	6,789.3	4,722.4	4,736.0
Model 6	6,774.2	6,785.3	4,720.3	4,734.6
Model 7	6,777	6,789.4	4,718.9	4,733.3
Model 8	6,777.8	6,790.1	4,720.5	4,734.9
Model 9	6,776.2	6,787.7	4,722.3	4,736.6

Subset is the dataset excluding the two species showing negative density dependence in the species-level analysis. Bold values denote significant effects (*P* < 0.05). AIC values are presented for each of the nine models compared (see Table 2)

*BF* best-fit model, *n* number of seedlings used in the analysis, *SP* number of seedling species used in the analysis

However, after removing the two species that showed negative density dependence in the species-level analysis (*Deutzia glabrata* and *Philadelphus schrenkii*), community-wide seedling survival was best fitted by model 4, indicating that there was an effect of overall basal area of trees ≥1 cm dbh, but no effect of seedling neighbors. The effect of overall basal area of trees ≥1 cm dbh remained significantly positive (Table 3).

Controlling for habitat heterogeneity did not qualitatively alter the observed patterns of conspecific negative density dependence at the seedling stage. Including canopy openness and topographic position as covariates in the models did change the best-fit models for 6 of the 11 focal species (Online Resource 6 vs. 5). However, for all 6 of those species, the best-fit models did not include separate terms for conspecific and heterospecific neighbors, regardless of whether the model controlled for habitat heterogeneity. For the community-level analyses, the best-fit models did not change when including canopy openness and topographic position as covariates, and the coefficient values for neighbor effects were similar compared with models that did not include these habitat variables (Table 3).

### Later-stage conspecific density dependence

We calculated the number of species showing aggregated, random and regular patterns at the sapling and juvenile stage at each (1 m) scale up to 10 m. For saplings, 11 of 15 species exhibited additional aggregation relative to adults (i.e. saplings were more clustered than adults), 7 species showed random patterns (i.e. not significantly different from the adults), and 1 species showed more regular patterns (i.e. saplings were less aggregated than the adults) at scales up to 10 m (Fig. 1d). For juveniles, 8 of 15 species exhibited additional aggregation relative to adults, 13 species showed random patterns, and no species showed more regular patterns up to 10 m (Fig. 1e).

The 11 species (73 %) that exhibited additional aggregation relative to adults in the sapling stage were all found to have a decline in the strength of additional clustering from the sapling to juvenile stage at the scale of 0–10 m, indicating that the majority of abundant species showed conspecific density dependence across the study area during the sapling to juvenile transition (Table 4). For the 4 species that did not exhibit additional aggregation relative to adults in the sapling stage at the scale of 0–10 m (*Acanthopanax senticosus*, *Corylus mandshurica*, *Philadelphus schrenkii* and *Syringa reticulata* var. *mandshurica*), we cannot test whether they experienced density dependence from the sapling to juvenile stage with our methods. However, since saplings were not more aggregated than adult trees for these species, it is unlikely that conspecific density-dependent thinning occurred beyond the sapling stage in these species.

**Table 4:** Values of conspecific thinning, *d*(*r*), for the 11 species that exhibited thinning effects from the sapling to juvenile stage at the scale of 0–10 m when habitat heterogeneity was factored out

Scale *r* (m)	0	1	2	3	4	5	6	7	8	9	10
*Abies nephrolepis*	**29.2**	16.5	12.7	6.5	3.9	2.3	1.7	0.7	0.4	–	–
*Acer mono*	**0.9**	0.2	0.2	0.1	0.2	0.2	0.1	0.0	0.1	–	0.0
*Acer tegmentosum*	–	–	–	0.1	0.6	0.7	**0.8**	0.6	0.2	0.1	–
*Acer ukurunduense*	–	–	–	–	0.2	**0.3**	–	–	–	–	–
*Betula costata*	**17.8**	17.2	12.9	9.5	6.6	4.9	4.2	4.2	3.3	3.2	2.2
*Euonymus pauciflorus*	–	1.1	**1.2**	–	–	–	–	–	–	–	–
*Fraxinus mandshurica*	1.2	–	0.2	3.7	4.6	**4.6**	3.8	3.0	1.6	1.2	0.6
*Pinus koraiensis*	**58.1**	36.4	27.6	17.5	11.6	10.5	10.6	10.2	8.0	6.9	5.3
*Tilia amurensis*	**1.3**	–	–	–	–	–	–	–	–	–	–
*Tilia mandshurica*	–	–	–	–	8.4	**11.9**	11.4	6.9	–	–	–
*Ulmus laciniata*	**3.7**	3.1	1.7	0.3	–	–	0.1	–	0.0	0.2	0.4

Bold values denote the maximum strength of conspecific thinning (*d*_max_) at the scale of 0–30 m

The number of species showing conspecific density-dependent thinning decreased with increasing spatial scale (Fig. 1f). Six of the 11 species that exhibited conspecific density-dependent thinning at the scale of 0–10 m reached a maximum strength of thinning at the scale of 0 m (in a 1 × 1 m grid cell) (Table 4). Furthermore, the largest radius at which maximum thinning occurred was only 6 m (for *Acer tegmentosum*; Table 4). The thinning intensity also had a trend of decreasing with increasing scales for most species (Table 4). Together, these results imply that conspecific density-dependent thinning occurred predominantly at close distances among neighbors.

The above analyses accounted for habitat heterogeneity by using mature tree distributions as controls. When habitat heterogeneity was not controlled for, we found an increase in the number of species showing conspecific thinning at larger scales (>20 m) (Online Resource 7c). As a result, we did not see a decrease in the number of species showing conspecific density-dependent thinning with increasing spatial scale, as was found when controlling for habitat heterogeneity. Nonetheless, there was no difference in the overall number of species (11 of 15 focal species) found to have a decline in the strength of additional aggregation relative to the randomized adult locations from sapling to juvenile stage at the scale of 0–10 m (Online Resource 8). In other words, the same number of species exhibited conspecific density-dependent thinning at local (<10 m) scales regardless of whether habitat heterogeneity was controlled for; however, there were differences in the individual species exhibiting conspecific thinning. *Tilia amurensis*, which exhibited conspecific density-dependent thinning from the sapling to juvenile stage when habitat heterogeneity was factored out, did not show that trend when habitat heterogeneity was unaccounted for. *Philadelphus schrenkii*, which did not show conspecific density-dependent thinning when habitat heterogeneity was factored out, was found to exhibit conspecific density-dependent thinning when habitat heterogeneity was not accounted for (Online Resource 8; Table 4). In addition, for species that showed significant conspecific thinning regardless of whether habitat heterogeneity was accounted for, there were often differences in the scale of maximum conspecific thinning. For example, *Acer ukurunduense* and *Tilia mandshurica* reached their maximum strength of conspecific thinning, *d*_max_, at scales of *r* > 15 m when habitat heterogeneity was not accounted for, but both reached *d*_max_ at the scale of 5 m when habitat heterogeneity was accounted for (Online Resource 8; Table 4).

## Discussion

Density dependence has been hypothesized to be one of the most prominent mechanisms contributing to the maintenance of diversity (Janzen 1970; Connell 1971; Hooper 1998; Chesson 2000; Volkov et al. 2005). Though many studies have examined how this mechanism operates, most have focused on a single life-history stage (e.g., Bell et al. 2006; Diez 2007; Queenborough et al. 2009) and few studies have factored out the potentially confounding influence of habitat heterogeneity (but see He and Duncan 2000; Zhu et al. 2010; Chen et al. 2010). As far as we know, this is the first study of density dependence in trees to include more than one life-history stage while controlling for habitat heterogeneity. Our study of the impact of the biotic neighborhood on the established seedling and sapling to juvenile transition stages showed that conspecific neighbors tend to have a negative impact on survival. In addition, our analyses demonstrate the influence of habitat heterogeneity on analyses of density dependence.

### Habitat heterogeneity

The increase in establishment driven by favorable habitat may offset the thinning of conspecific trees due to negative density dependence (Wright 2002). Our results demonstrate the importance of controlling for habitat heterogeneity when testing for density dependence in plant communities. For both early and later life stages, we compared the results with and without controlling for habitat heterogeneity. For the seedling stage, we found that controlling for habitat heterogeneity did not qualitatively alter our results of negative density dependence at the community level or the proportion of focal species exhibiting negative density-dependent seedling survival. At the seedling stage, survival may be more strongly affected by conspecific density than abiotic conditions. However, our models only included canopy openness and topographic position to control for possible habitat preferences. Therefore, we cannot rule out the possibility that unmeasured habitat variables, such as soil nutrients and temperature, may be masking patterns of density dependence. For the sapling to juvenile stage, the same number of species showed density dependence when controlling and not controlling for habitat heterogeneity. However, several species showed differing patterns in terms of the significance or scale of density dependence when factoring out habitat heterogeneity. When habitat heterogeneity was not accounted for, conspecific thinning tended to be detected at larger spatial scales, likely driven by unfavorable habitat and not tree–tree interactions. Previous studies have also pointed out the confounding effects of habitat heterogeneity on density dependence analysis. For example, He and Duncan (2000) tested intra- and interspecific density-dependent effects on survival of three species in an old-growth Douglas fir (*Pseudotsuga menziesii*) forest, where elevation gradients control local variation in site conditions. They found that, after controlling for elevation, the probability of western hemlock (*Tsuga heterophylla*) survival was no longer significantly higher in less dense patches of Douglas fir. Zhu et al. (2010), in a study of 47 abundant species in a subtropical forest, also used the random labeling method and found that the number of species showing density dependence was different when factoring out habitat heterogeneity. In a study of density-dependent seedling survival in a subtropical forest, Chen et al. (2010) found that habitat heterogeneity explained more variation among species in seedling survival than species abundance, and concluded that tests for community-level consequences of density dependence must account for habitat heterogeneity. Those findings, together with our study, indicate that failing to consider habitat heterogeneity could lead to incorrect inferences about density-dependent effects, and therefore controlling for habitat heterogeneity is necessary in studies of density dependence.

### Density dependence across multiple life stages

In this study, we analyzed density dependence for both earlier (the established seedling stage) and later life-history stages (the sapling to juvenile transition) of tree species in the Lianghsui FDP. For seedlings, we found a significant negative effect of local conspecific seedling density on survival when analyzing all species in the community together, consistent with predictions of the Janzen–Connell hypothesis. However, in separate species-level analyses, we detected negative effects of conspecific seedling neighbors on focal seedling survival for only two species, *Deutzia glabrata* (which accounted for 22.1 % of total seedlings) and *Philadelphus schrenkii* (which accounted for 8.7 % of total seedlings). This suggests that our community level finding of density dependence was likely driven by these two abundant species. Indeed, when we removed these species from the dataset, we did not detect significant conspecific density dependence. Thus, our results emphasize how community-level analyses can conceal variation among species in density dependence. This is consistent with recent studies that have found wide variation among species in the strength of density dependence (Comita et al. 2010; Kobe and Vriesendorp 2011).

Relatively few studies of density dependence have been conducted for saplings and larger trees at the community level (Carson et al. 2008). In this study, we examined density dependence across the sapling-to-juvenile transition. At this later stage, we found that 11 of 15 focal species showed conspecific density-dependent thinning. For those 11 species, we found that the thinning occurred predominantly at very small scales: the maximum strength of thinning occurred only up to 6 m, and 6 species reached a maximum strength of thinning at the scale of <1 m. This may due to the decline in aggregation of saplings with increasing distance from parent trees, likely caused by dispersal limitation (Hubbell and Foster 1983; He et al. 1997). The observed local-scale conspecific thinning could have resulted from strong intraspecific competition for resources or host-specific natural enemy attack leading to density-dependent mortality. At the smallest spatial scales, we also cannot rule out the possibility that thinning was caused by physical space constraints.

Studies of density dependence at later life stages in other forests have similarly found that the majority of species tested exhibit significant conspecific density dependence (e.g., Wills et al. 1997; Peters 2003; Stoll and Newbery 2005; Zhang et al. 2009). For example, Wills et al. (1997) found that for 67 of 84 focal species in Panama, recruitment of ≥1 cm dbh saplings was negatively correlated with conspecific basal area in at least one quadrat size, and intraspecific effects were stronger than interspecific effects. Similarly, in a study of neighbor effects on saplings and trees (dbh ≥ 1 cm), Peters (2003) found evidence of density-dependent mortality for saplings and trees of >75 % of the species tested at sites in Pasoh, Malaysia, and BCI, Panama. These studies, together with the results presented here, show that density-dependent effects can be prevalent at later life stages and should not be ignored.

Nonetheless, density dependence is often found to be more prevalent at earlier compared to later life stages in tree communities (e.g., Hille Ris Lambers et al. 2002; Comita and Hubbell 2009). This pattern may occur if density dependence during earlier stages is sufficiently strong to thin out conspecifics to levels below which negative effects of density are not detectable at later stages. However, we found significant negative conspecific density effects for only 2 of the 11 abundant species in our analysis of density-dependent survival at the established seedling stage, but significant conspecific density-dependent thinning from the sapling to juvenile stage for 11 of the 15 focal species, including 4 of the species that did not show density dependence effects at the seedling stage. Thus, our results do not support the idea that density dependence is stronger at earlier stages. However, the higher incidence of density dependence at later stages in our study may reflect the different methods used to analyze density dependence at earlier and later life stages. We examined density dependence at later life-history stages using spatial pattern analysis, which takes advantage of the accumulated effects of density-dependent mortality over time as individuals of a species move from one size class to another. In contrast, for seedlings, we used direct observations of seedling mortality over 5 years, which may not have been sufficient to detect density dependence for some species.

The prevalence of density-dependent mortality found in our study has important implications beyond our understanding of the forces that structure this particular temperate forest. Janzen–Connell effects were assumed to be stronger in tropical than temperate forests because of higher numbers of specialist predators and pathogens in the former (Janzen 1970; Connell 1971; Coley and Barone 1996; Givnish 1999; Harms et al. 2000; Dyer et al. 2007), and the latitudinal gradient in tree diversity has been hypothesized to be caused by decreasing Janzen–Connell effects with increasing latitude. However, studies have found that density-dependent mortality is as common in temperate forests as in tropical forests. For example, Hille Ris Lambers et al. (2002) found the proportion of species affected by negative density dependence at the seed and seedling stages in a North American temperate forest was equivalent to that reported for tropical forests, though they acknowledged that the magnitude of density-dependent effects may be higher in tropical forests. Our study of density dependence in Liangshui FDP also supports the idea that density dependence may be as important in temperate forests as in tropical forests, and density dependence alone is unlikely to explain latitudinal tree diversity differences in the world’s forests (but see Johnson et al. 2012).

## Conclusion

In combination with previous studies from tropical and subtropical forests, our results from an old-growth temperate forest indicate that significant negative effects of local conspecific density occur at multiple life stages in tree communities. This suggests that density dependence plays an important role in enhancing community diversity of forests in different latitudes. Although we found negative conspecific effects at multiple stages, species exhibiting negative density dependence at one life stage did not necessarily exhibit it at other stages. Thus, analyses that focus on a single life stage may underestimate the prevalence and importance of density dependence in tree communities. Similarly, our results suggest that studies that fail to take into account confounding factors, such as habitat heterogeneity and species-level variation, may also mischaracterize the role of density dependence in shaping plant communities. Therefore, we recommend that future studies take habitat heterogeneity and other potentially confounding factors into account and also test for effects of conspecific neighbors across all life stages.

## Electronic supplementary material

Below is the link to the electronic supplementary material.

Supplementary material 1 (DOCX 703 kb)
