# Supplementary material for: Density dependence across multiple life stages in a temperate old-growth forest of northeast China
Source: Oecologia. 2012 Oct 2;172(1):207–17. doi: 10.1007/s00442-012-2481-y (PMC3627022; doi:10.1007/s00442-012-2481-y)
Supplement: Supplementary file 1 — Supplementary material 1 (DOCX 703 kb) [file 442_2012_2481_MOESM1_ESM.docx]

**Density dependence across multiple life stages in a temperate old-growth forest of northeast China**

**Tiefeng Piao** **• Liza S. Comita** **• Guangze Jin** **• Ji Hong Kim**

G. Z. Jin ([taxus@126.com](mailto:taxus@126.com))

Center for Ecological Research, Northeast Forestry University, Harbin, 150040, China.

Phone number: + 86 451 82191823

**Electronic Supplemental Material**

**ESM_1** The location and contour map of the 9-ha Liangshui forest dynamic plot (Liangshui FDP), China. Numbers denote altitude (m), the unit of (x, y) – axes is meters, and points represent individual adults (*dbh* > 15cm) of the dominant tree species *Pinus koraiensis*

**ESM_2** List of the 15 focal species and the number of individuals in the sapling, juvenile and adult stages for each species in the Liangshui forest dynamic plot. S, US, UL, CS and CL denote shrubs, small understory tree species, large understory tree species, small canopy tree species, and large canopy tree species, respectively

| Species | Family | Growth  form | Number of individuals | | | |
| --- | --- | --- | --- | --- | --- | --- |
|  |  |  | Sapling | Juvenile | Adult | Total |
| *Abies nephrolepis* | *Pinaceae* | CS | 178 | 116 | 601 | 895 |
| *Acanthopanax senticosus* | *Araliaceae* | S | 1132 | 534 | 300 | 1966 |
| *Acer mono* | *Sapindaceae* | UL | 777 | 578 | 808 | 2163 |
| *Acer tegmentosum* | *Sapindaceae* | UL | 409 | 319 | 274 | 1002 |
| *Acer ukurunduense* | *Sapindaceae* | US | 332 | 411 | 547 | 1290 |
| *Betula costata* | *Betulaceae* | CS | 208 | 187 | 238 | 633 |
| *Corylus mandshurica* | *Betulaceae* | S | 1175 | 1215 | 2439 | 4829 |
| *Euonymus pauciflorus* | *Celastraceae* | S | 509 | 405 | 368 | 1282 |
| *Fraxinus mandshurica* | *Oleaceae* | CL | 223 | 64 | 121 | 408 |
| *Philadelphus schrenkii* | *Saxifragaceae* | S | 375 | 254 | 181 | 810 |
| *Pinus koraiensis* | *Pinaceae* | CL | 104 | 59 | 1040 | 1203 |
| *Syringa reticulata* var. *mandshurica* | *Oleaceae* | US | 163 | 161 | 278 | 602 |
| *Tilia amurensis* | *Malvaceae* | CS | 303 | 180 | 271 | 754 |
| *Tilia mandshurica* | *Malvaceae* | UL | 63 | 88 | 87 | 238 |
| *Ulmus laciniata* | *Ulmaceae* | CS | 632 | 147 | 206 | 985 |

**ESM_3** Spatial autocorrelation analysis of model residuals (based on the full model) for the analysis of seedling survival in the Liangshui FDP. Confidence intervals were generated by 999 simulations

**ESM_4** Detection of large-scale habitat heterogeneity for the 15 focal species in the Liangshui FDP, using the L-function under homogeneous Poisson null model

**ESM_5** Effects of conspecific and heterospecific seedling and adult neighbors on five-year survival of established seedlings of 11 abundant tree species (N > 99), without controlling for habitat preference. Bold values denote significant effects (*P* < 0.05). Models were compared using Akaike’s Information Criterion (AIC). - signifies that the parameter was not included in the best-fit model

| Species | AS | AM | AT | CM | DG | EP | FM | LC | PS | RM | SR |
| --- | --- | --- | --- | --- | --- | --- | --- | --- | --- | --- | --- |
| Number of individuals | 639 | 199 | 258 | 535 | 1272 | 474 | 193 | 739 | 503 | 188 | 101 |
| Best-fit model | 1 | 4 | 1 | 4 | 9 | 1 | 1 | 1 | 5 | 4 | 2 |
| Paremeters values |  | | | | | | | | | | |
| *H* | 0.139 | 0.324 | 0.143 | **0.321** | **0.358** | **0.340** | -0.344 | **0.294** | **0.349** | 0.080 | 1.471 |
| *S_CON_* | - | - | - | - | **-0.171** | - | - | - | **-0.269** | - | - |
| *S_HET_* | - | - | - | - | 0.008 | - | - | - | 0.025 | - | - |
| *S_TOTAL_* | - | - | - | - | - | - | - | - | - | - | 0.476 |
| *B_CON_* | - | - | - | - | **-0.262** | - | - | - | - | - | - |
| *B_HET_* | - | - | - | - | **0.168** | - | - | - | - | - | - |
| *B_TOTAL_* | - | 0.292 | - | 0.167 | - | - | - | - | - | 0.331 | - |
| AIC |  | | | | | | | | | | |
| Model 1 | 790.9 | 205.3 | 300.0 | 663.3 | 1534.8 | 458.7 | 241.8 | 936.7 | 560.7 | 245.8 | 54.9 |
| Model 2 | 792.8 | 205.4 | 301.8 | 665.1 | 1536.8 | 460.6 | 243.1 | 936.7 | 562.3 | 247.5 | 54.0 |
| Model 3 | 794.8 | 205.7 | 301.9 | 664.6 | 1534.5 | 462.0 | 242.6 | 937.6 | 563.8 | 247.3 | 55.1 |
| Model 4 | 792.8 | 204.7 | 300.4 | 663.2 | 1533.0 | 460.0 | 242.0 | 938.1 | 562.4 | 245.3 | 54.2 |
| Model 5 | 794.8 | 207.1 | 303.7 | 667.1 | 1534.4 | 460.6 | 245.1 | 938.3 | 559.7 | 247.3 | 56.0 |
| Model 6 | 796.8 | 207.4 | 303.8 | 666.6 | 1533.4 | 461.7 | 244.6 | 939.0 | 560.8 | 247.8 | 57.0 |
| Model 7 | 793.8 | 206.6 | 302.2 | 664.5 | 1522.2 | 461.8 | 243.4 | 939.2 | 563.7 | 246.2 | 57.8 |
| Model 8 | 795.8 | 207.7 | 303.7 | 665.8 | 1523.4 | 463.8 | 244.0 | 938.5 | 564.9 | 248.2 | 56.9 |
| Model 9 | 797.8 | 209.3 | 305.6 | 667.8 | 1521.7 | 463.2 | 245.9 | 939.8 | 561.9 | 248.8 | 63.4 |

AS: *Acanthopanax senticosus*, AM: *Acer mono*, AT: *Acer tegmentosum*, CM: *Corylus mandshurica*, DG: *Deutzia glabrata*, EP: *Euonymus pauciflorus*, FM: *Fraxinus mandshurica*, LC: *Lonicera chrysantha*, PS: *Philadelphus schrenkii*, RM: *Ribes mandshuricum*, SR: *Syringa reticulata* var. *mandshurica*.**ESM_6** Effects of conspecific and heterospecific seedling and adult neighbors on five-year survival of established seedlings of 11 abundant tree species (N > 99), with canopy openness and topography position included as covariates in the models controlling for habitat preference. Bold values denote significant effects (*P* < 0.05). Models were compared using Akaike’s Information Criterion (AIC). - signifies that the parameter was not included in the best-fit model

| Species | AS | AM | AT | CM | DG | EP | FM | LC | PS | RM | SR |
| --- | --- | --- | --- | --- | --- | --- | --- | --- | --- | --- | --- |
| Number of individuals | 639 | 199 | 258 | 535 | 1272 | 474 | 193 | 739 | 503 | 188 | 101 |
| Best-fit model | 1 | 1 | 1 | 1 | 9 | 4 | 2 | 1 | 5 | 1 | 1 |
| Paremeter values |  | | | | | | | | | | |
| *Canopy open* | 0.067 | **0.595** | 0.062 | -0.073 | 0.016 | **-0.473** | -0.099 | 0.093 | -0.155 | -0.390 | -0.562 |
| *TOPO*: upper slope | -0.980 | -14.087 | -14.690 | 0.172 | **-2.673** | -0.197 | -15.100 | -15.098 | -12.858 | -0.177 | - |
| *TOPO*: lower slope | -0.864 | -14.246 | -15.534 | -0.219 | **-2.831** | -0.519 | -14.602 | -14.866 | -13.111 | -0.249 | -15.198 |
| *TOPO*: valley | -0.138 | -15.343 | -16.305 | 0.293 | **-3.013** | -1.044 | -15.837 | -14.279 | -12.158 | 0.004 | 0.751 |
| *H* | 0.140 | 0.361 | 0.157 | **0.336** | **0.353** | **0.550** | -0.279 | **0.293** | **0.319** | 0.101 | 1.321 |
| *S_CON_* | - | - | - | - | **-0.185** | - | - | - | **-0.273** | - | - |
| *S_HET_* | - | - | - | - | -0.046 | - | - | - | 0.031 | - | - |
| *S_TOTAL_* | - | - | - | - | - | - | -0.258 | - | - | - | - |
| *B_CON_* | - | - | - | - | **-0.282** | - | - | - | - | - | - |
| *B_HET_* | - | - | - | - | 0.145 | - | - | - | - | - | - |
| *B_TOTAL_* | - | - | - | - | - | -0.203 | - | - | - | - | - |
| AIC values |  | | | | | | | | | | |
| Model 1 | 792.7 | 200.4 | 300.3 | 667.7 | 1529.3 | 449.3 | 241.6 | 935.3 | 559.9 | 250.3 | 57.5 |
| Model 2 | 794.6 | 202.2 | 300.6 | 669.6 | 1530.6 | 451.3 | 241.6 | 935.4 | 561.6 | 252.2 | 59.3 |
| Model 3 | 796.5 | 203.5 | 301.9 | 670.0 | 1529.5 | 450.6 | 243.2 | 936.1 | 562.7 | 252.3 | 64.2 |
| Model 4 | 794.5 | 201.6 | 302.0 | 668.5 | 1529.0 | 448.6 | 243.5 | 936.6 | 561.3 | 250.3 | 62.8 |
| Model 5 | 796.6 | 204.0 | 302.5 | 671.5 | 1528.9 | 452.6 | 243.5 | 936.8 | 558.7 | 251.0 | 62.9 |
| Model 6 | 798.5 | 205.3 | 303.9 | 672.0 | 1528.7 | 451.9 | 245.0 | 937.3 | 559.4 | 251.8 | 66.2 |
| Model 7 | 795.8 | 203.6 | 302.8 | 669.3 | 1516.6 | 450.0 | 244.9 | 937.4 | 562.9 | 250.8 | 64.5 |
| Model 8 | 797.8 | 205.5 | 302.5 | 670.7 | 1516.0 | 452.0 | 244.6 | 936.4 | 564.1 | 252.8 | 65.9 |
| Model 9 | 799.8 | 207.3 | 304.4 | 672.7 | 1514.8 | 453.0 | 246.5 | 937.5 | 560.7 | 252.2 | 67.9 |

**ESM_7** Analysis of density dependence without controlling for habitat heterogeneity for the 15 focal species a) saplings and b) juveniles as cases. Solid circles: number of species showing the test statistic *g_ij_*(*r*) - *g_ii_*(*r*) < 0, open circles: *g_ij_*(*r*) - *g_ii_*(*r*) > 0 and open squares: *g_ij_*(*r*) - *g_ii_*(*r*) = 0. c) Number of focal species showing density dependence at each scale

**ESM_8** Values of *d(r)* for the 11 species that exhibited conspecific density dependent thinning from sapling to juvenile stage at the scales of 0-10 m when habitat heterogeneity was not factored out. *d_max_*(*r*) is the scale at which maximum strength of conspecific thinning occurred from the sapling to juvenile stage within the scale range 0-30 m

| Scale *r* (m) | 0 | 1 | 2 | 3 | 4 | 5 | 6 | 7 | 8 | 9 | 10 | *d_max_*(*r*) |
| --- | --- | --- | --- | --- | --- | --- | --- | --- | --- | --- | --- | --- |
| *Abies nephrolepis* | 27.1 | 15.2 | 11 | 5.3 | 3 | 1.7 | 1.1 | 0.2 | 0 | - | - | 0 |
| *Acer mono* | 1.2 | 0.2 | 0.2 | 0.1 | 0.1 | 0 | 0 | - | 0.1 | 0 | 0.1 | 0 |
| *Acer tegmentosum* | - | - | - | - | - | - | 0.4 | 0.5 | 0.4 | 0.3 | 0.2 | 7 |
| *Acer ukurunduense* | - | - | - | - | - | 0 | - | - | - | - | - | 26 |
| *Betula costata* | 11.1 | 11.8 | 7.4 | 4.4 | 1.8 | 0.4 | - | - | - | - | - | 1 |
| *Euonymus pauciflorus* | 1.6 | 0.1 | 0.6 | 0.3 | 0.5 | 0 | 0 | - | - | - | - | 0 |
| *Fraxinus mandshurica* | - | - | - | - | 2.2 | 3.9 | 3.1 | 2 | 0.7 | 0.2 | - | 5 |
| *Philadelphus schrenkii* | - | 0.3 | 0.6 | 0.6 | 0.2 | 0.3 | 0.5 | 0.6 | 0.2 | - | - | 7 |
| *Pinus koraiensis* | 56.6 | 35.5 | 26.5 | 16.3 | 10 | 9.1 | 9.3 | 9.1 | 6.9 | 5.8 | 4.3 | 0 |
| *Tilia mandshurica* | - | - | - | - | - | - | 0.3 | - | - | - | - | 18 |
| *Ulmus laciniata* | 3.7 | 2.6 | 1.1 | - | - | - | - | - | - | - | 0 | 0 |
